# 13-year-old tuberous sclerosis patient with renal cell carcinoma associated with multiple renal angiomyolipomas developing multifocal micronodular pneumocyte hyperplasia

**DOI:** 10.1186/1472-6890-13-4

**Published:** 2013-02-05

**Authors:** Carl Ludwig Behnes, Gunther Schütze, Christoph Engelke, Felix Bremmer, Bastian Gunawan, Heinz-Joachim Radzun, Stefan Schweyer

**Affiliations:** 1 Department of Pathology, University Medicine Göttingen, Robert-Koch-Str. 40, Göttingen, D-37075, Germany; 2 Department of Neuroradiology, University Medicine Göttingen, Robert-Koch-Str. 40, Göttingen, D-37075, Germany; 3 Department of Diagnostic Radiology, University Medicine Göttingen, Robert-Koch-Str. 40, Göttingen, D-37075, Germany

**Keywords:** Multifocal micronodular pneumocyte hyperplasia (MMPH), Renal cell carcinoma, Angioleiomyolipoma, Tuberous sclerosis complex

## Abstract

****Background**:**

The autosomal dominant tumor syndrome tuberous sclerosis complex is caused by the mutated TSC1 gene, *hamartin*, and the TSC2 gene, *tuberin*. Patients with this complex develop typical cutaneus symptoms such as *peau chagrin* or angiofibromas of the skin as well as other lesions such as astrocytomas in the brain and lymphangioleiomyomatosis in the lung. Only a few tuberous sclerosis patients have been described who showed a multifocal micronodular pneumocyte hyperplasia of the lung. Another benign tumor which often occurs together with tuberous sclerosis is the angiomyolipoma of the kidney. Furthermore, an increased incidence of renal cell carcinoma in connection with tuberous sclerosis has also been proven.

****Case presentation**:**

We report a 13-year-old white girl with epilepsy and hypopigmented skin lesions. Radiological studies demonstrated the typical cortical tubers leading to the diagnosis of tuberous sclerosis. In the following examinations a large number of angiomyolipomas were found in both kidneys. One lesion showed an increasing size and tumor like aspects in magnetic resonance imaging. The pathological examination of the following tumorectomy demonstrated an unclassified renal cell carcinoma. Four months postoperatively, a follow-up computer tomography revealed multiple bilateral pulmonary nodules. To exclude lung metastases of the renal cell carcinoma, multiple open-lung biopsies were performed.

****Conclusion**:**

Here we report a diagnostically challenging case of a 13-year-old patient with tuberous sclerosis and angiomyolipomas of the kidney who developed an unclassified renal cell carcinoma as well as multifocal micronodular pneumocyte hyperplasia.

## Background

The TSC1 gene, *hamartin*, and the TSC2 gene, *tuberin*, were identified in 1997 and 1993, respectively. These tumor suppressor genes are mutated in the autosomal dominant tumor syndrome tuberous sclerosis complex (TSC) [1-3]. TSC is characterized by the formation of hamartomatous lesions in multiple organ systems. Besides the typical skin manifestations, e.g. cutaneous angiofibroma, *peau chagrin*, or subungual fibromas, TSC patients develop numerous brain lesions such as cortical tubers and subependymal giant cell astrocytomas. In addition, patients show an increased incidence of benign tumors such as multiple renal angiomyolipomas (AMLs), lymphangioleiomyomatosis (LAM) of the lungs, cardiac rhabdomyomas, and adenomatous polyps of the duodenum and the small intestine. Another rare manifestation of TSC is the so-called multifocal micronodular pneumocyte hyperplasia (MMPH), first recognized in 1991 by HH Popper [4]. TSC is also associated with an increased incidence of renal cell carcinomas (RCC) [5].

In this report, we present a case of a young patient with TSC and multiple AMLs who developed a RCC as well as MMPH. We discuss the histological, immunohistological and molecular features of these lesions with emphasis on the diagnostic requirements and differential diagnosis.

## Case presentation

### Clinical findings

**Figure 1:**
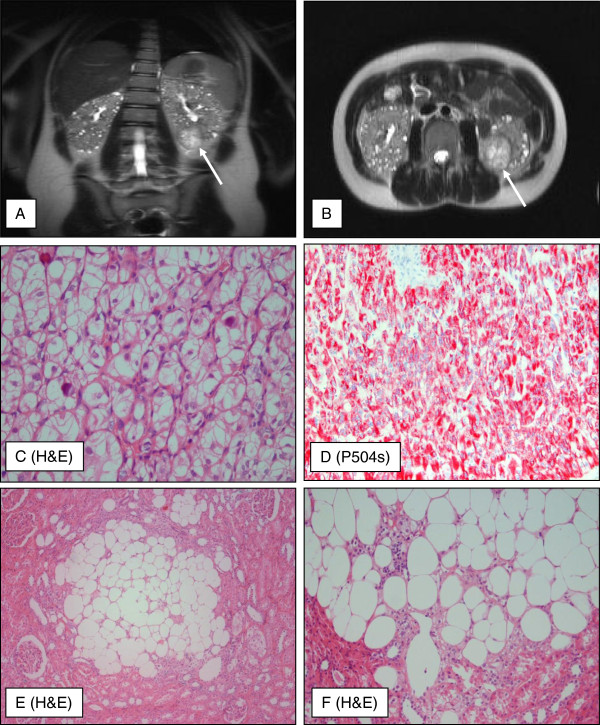
**Morphology of renal tumors.** A coronary (**A**) and axial (**B**) magnetic resonance imaging (MRI) scan of the kidneys showing multiple cysts and a tumor in the left kidney measuring 3.5 cm in diameter. Histologically, the renal cell carcinoma was composed of solid and nested tumor cells with voluminous clear cytoplasm (**C**, x200) and strong expression of P504s (**D,** x100). The AMLs demonstrated the pathognomonic components of this tumor entity, mature fat and blood vessels (**E,** x40 / **F,** x100).

**Figure 2:**
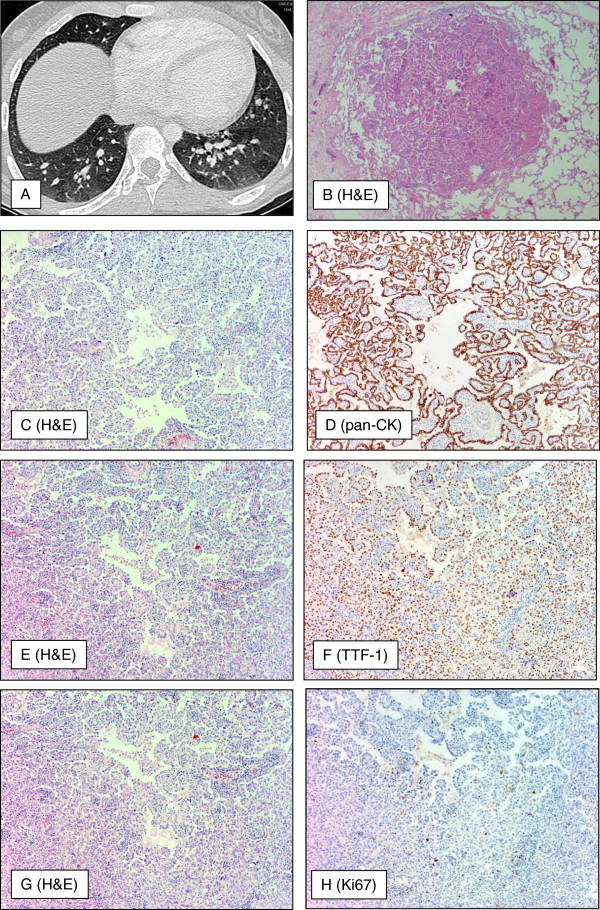
**Morphology of pulmonary lesions.** Axial (**A**) computer tomography (CT) of the chest revealed multiple soft tissue density nodules of 1–6 mm in diameter in both lungs with a minimal peripheral ground glass component in a random peribronchovascular-centrilobular distribution pattern. There was slight size predominance in the lower lung zones with the largest nodules being located in the middle and lower lobes. Furthermore, there was moderate regional air trapping in the basal lower lobes. Histologically and immunohistologically, nodules were composed of proliferated and enlarged type II pneumocytes (**B,** x40 /**C**, **E**, **G** x100) expressing pan-CK (**E,** x100), TTF-1 (**F,** x100) with low proliferation rate in Ki67 (**H**, x100).

The patient was a 13-year-old Caucasian girl who suffered from epilepsy in the form of salaam seizures that had begun at the age of 6 months. Aside from the seizures, clinical examination revealed hypopigmented skin lesions on the face and abdomen which were classified as *peau chagrin*. In addition, radiologic studies demonstrated the typical cortical tubers which led to the diagnosis of TSC. In the annual follow-up routine examinations, a large number of blurred coin lesions in both kidneys were found which were classified as multiple AMLs in the context of TSC. At the age of 13, one coin lesion showed an increase in size and magnetic resonance imaging (MRI) demonstrated a 3.5-cm tumor in the left kidney (Figure 1A and B), upon which the the patient underwent tumorectomy of that kidney. The postoperative period was uneventful and the patient was discharged 7 days after surgery. Four months postoperatively, a follow-up CT scan of the chest revealed multiple bilateral pulmonary nodules measuring 1–6 mm (Figure 2A). Due to the RCC diagnosis, and also to rule out lung metastases, follow-up chest CT scans were performed after 6 and 9 months, showing an unchanged status of the pulmonary nodules in terms of size and number, with CT-morphological appearances typical of MMPH.One year after the renal tumor resection, multiple open lung biopsies of both lungs were performed to obtain histological evidence. After an uneventful recovery the patient was discharged after 5 days. 

### Pathological findings of the renal tumors

Macroscopically, the tumorectomy revealed a 5 cm yellowish red tumor with multiple 1 to 2 mm yellow nodules in the adjacent kidney tissue.

The 5 cm tumor of the kidney showed a solid and nested, sometimes papillary architecture, and was composed of clear cells with voluminous cytoplasm, discrete cell borders, and vesicular chromatin. In addition, psammomatous calcification and hyaline nodules could be detected (Figure 1C). Immunohistochemical examinations revealed a weak to strong staining for vimentin and CD10 in more than 50% of neoplastic cells. All tumor cells showed a strong staining for P504s (Figure 1D). Pan-CK was only weakly expressed in scattered tumor cells. CK7 or HMB-45 could not be shown in the carcinoma cells. Based on the morphological and immunohistochemical findings, it was hypothesized that the tumor might be a RCC of XP 11.2 translocation type. This hypothesis was confirmed by three independent consultant pathologists (Leuschner I, Department of Pathology, University of Kiel, Germany; Moch H, Department of Surgical Pathology, University of Zürich, Switzerland; and Argami P, Department of Pathology, The Johns Hopkins University, Baltimore, USA); however, a typical expression of TFE3 and TFEB could not be demonstrated. Comparative genome hybridization (CGH) analysis did not reveal chromosomal imbalances such as losses at 3p or gains at 17q specific for clear cell RCC or papillary RCC, respectively (data not shown).

Besides the 5 cm tumor, multiple 1 to 2 mm nodules were observed in the adjacent renal tissue, which showed the 3 components of AMLs and the typical immunohistochemically expression of HMB-45 (Figure 1E and F).

### Pathological findings of the lung tumors

Macroscopically, open-lung biopsies revealed 2 to 5 mm sharply demarcated and randomly distributed nodules in both lungs (Figure 2A).

The nodules were composed of proliferated and enlarged type II pneumocytes which had abundant eosinophilic cytoplasm and vesicular nuclei with prominent nucleoli and occasional eosinophilic inclusions. The cells formed ill-defined areas of papillae that filled the alveolar space and partly obscured the alveolar architecture. Significant atypia or mitosis could not be observed. The interalveolar septa were mild thickened with increased reticulin (Figure 2B, C, E, G). Immunohistochemically, the hyperplastic epithelial cells showed an expression of pan-CK, TTF-1 (Figure 2D, F), and EMA, whereas stains for HMB-45, actin, vimentin and ER were uniformly negative. The proliferation rate evaluated with MIB-1 was solely 2% (Figure 2H).

## Conclusion

TSC is an autosomal-dominant disorder characterized by a variably high incidence (90-95%) affecting 0.5-1 per 10,000 births. The diagnosis of TSC is currently made by using the criteria established by Gomez [6]. These criteria are composed of primary (e.g. subependymal nodes and facial angiofibroma), secondary, (e.g. AMLs and LAM), and tertiary features (e.g. hypomelanotic maculas). Whereas the hamartomas in TSC are benign lesions, other phakomatoses such as Von-Hippel-Lindau (VHL) disease are characterized by the development of malignant tumors. Therefore, RCC is a typical malignant tumor that is known to arise in VHL, whereas tuberous TSC patients also develop RCC, but only in rare cases [5]. The mechanisms behind the occurrence of RCC could be demonstrated in the Eker rat model which develops RCCs based on a mutation in TSC2 gene [7-9]. Furthermore, an unusual pulmonary manifestation of TSC is MMPH, typically presenting multiple pulmonary nodules on chest roentgenogram or CT scan [4,10,11].

The case described here shows the diagnostic challenge of young TSC patients who present with multiple AMLs of the kidney and develop an unclassified RCC. RCC often occur in young TSC patients [12,13] and demonstrate several subtypes [14]. In our case, in the follow-up months a CT scan revealed multiple nodules in the lungs. It is difficult to differentiate between benign lesions like LAM or MMPH and metastasis on the basis of CT scans only, as our present case demonstrates [15]. A definitive diagnosis of MMPH could only be made on the basis of histological examination of a lung biopsy. MMPH is extremely rare in TSC and in the literature less than 20 histologically documented cases are described and have been reported in association with LAM and AML [16]. One of these cases shows MMPH in a TSC patient who had undergone a bilateral nephrectomy because of RCC. AML are not reported in this case, but the authors also describe the difficulty they had differentiating between the benign MMPH and metastasis of the RCC [17].

The association between TSC mutation and RCC seems to be often underestimated in familiarly RCC [18]. Furthermore, the TSC 1/2 complex influences the beta-catenin pathway and could also play a functional role in other hereditary tumor syndromes such as Cowden’s disease or Peutz-Jeghers syndrome [19].

The case of a young TSC patient described here demonstrates a rare combination of AMLs, RCC and hamartomas in the lung, which posed a diagnostic dilemma.

## Consent

Written informed consent was obtained from the patient for publication of this case report and any accompanying images. A copy of the written consent is available for review by the Editor-in-Chief of this journal.

## Competing interests

The authors declare that they have no competing interests.

## Authors’ contributions

CLB and SS constructed the manuscript and carried out the pathological examination. GS and CE were responsible for the radiological data. BG participated in pathological and genetic examination. HJR and FB were responsible for critical revision of the manuscript and have been involved in drafting it. All authors read and approved the final manuscript.

## Pre-publication history

The pre-publication history for this paper can be accessed here:

http://www.biomedcentral.com/1472-6890/13/4/prepub
